# Validation of endogenous controls for gene expression studies in peripheral lymphocytes from war veterans with and without PTSD

**DOI:** 10.1186/1471-2199-11-26

**Published:** 2010-04-09

**Authors:** Jelena Brkljačić, Nikola Tanić, Danijela Vojnović Milutinović, Ivana Elaković, Sanja Manitašević Jovanović, Tatjana Perišić, Jadranka Dundjerski, Gordana Matić

**Affiliations:** 1 Department of Biochemistry, Institute for Biological Research "Siniša Stanković", University of Belgrade, 142 Despot Stefan Blvd., Belgrade, 11000, Serbia; 2 Department of Neurobiology, Institute for Biological Research "Siniša Stanković", University of Belgrade, 142 Despot Stefan Blvd., Belgrade, 11000, Serbia

## Abstract

**Background:**

Selection of appropriate endogenous control is a critical step in gene expression analysis. The aim of this study was to evaluate expression stability of four frequently used endogenous controls: β-actin, glyceraldehyde-3-phosphate dehydrogenase, β_2_-microglobulin and RNA polymerase II polypeptide A in peripheral blood mononuclear cells from war veterans with and without posttraumatic stress disorder (PTSD). The study was designed as to identify suitable reference gene(s) for normalization of gene expression in peripheral blood mononuclear cells in response to war trauma and/or PTSD.

**Results:**

The variability in expression of the four endogenous controls was assessed by TaqMan Real-time RT-PCR in peripheral blood mononuclear cells from: war veterans with current PTSD, those with lifetime PTSD, trauma controls and healthy subjects. Expression stability was analyzed by GeNorm and NormFinder software packages, and by direct comparison of Ct values. Both, GeNorm and NormFinder identified β-actin and glyceraldehyde-3-phosphate dehydrogenase as a pair of genes with the lowest stability value.

**Conclusions:**

The combination of β-actin and glyceraldehyde-3-phosphate dehydrogenase appeared to be the most suitable reference for studying alterations in gene expression in peripheral blood mononuclear cells related to vulnerability and resilience to PTSD, as well as to trauma-provoked developing of this disorder and recovery from it. Using glyceraldehyde-3-phosphate dehydrogenase, β-actin and β_2-_microglobulin as individual endogenous controls would provide satisfactory data, while RNA polymerase II polypeptide A could not be recommended.

## Background

Post-traumatic stress disorder (PTSD) is the most common war-related psychiatric disorder that could occur among war veterans and other people exposed to war-zone stress. Several studies investigated gene expression in peripheral blood mononuclear cells (PBMCs) from PTSD patients using microarray analysis [1-3], and Real-time PCR method [3,4]. Most of the data regarding gene expression in psychiatric disorders were obtained from studies carried out in PBMCs as easily available human tissue. It was previously shown that transcriptional changes in PBMCs reflect various pathological states [5,6]. Moreover, gene expression changes in PBMCs were paralleled by changes in neural tissue [7]. These arguments support exploiting blood lymphocytes as a source material for gene expression measurements in studying psychiatric disorders when brain tissue biopsy samples are unavailable [6-8].

Real-time PCR is a fast and convenient method for quantification of gene transcription, and is suitable for application both in research and clinical settings [9]. Compared to traditional methods for expression analysis, such as Northern blot, *in situ *hybridization, RNase protection assay and semi quantitative PCR, TaqMan Real-time PCR is considered the most reliable, since it does not include post PCR processing. It is simple, sensitive and accurate, and particularly suitable when working with small amounts of starting material [10]. Therefore, Real-time PCR has been a method of choice for investigation of gene expression in various diseases. However, the reliability of results depends on precision of many steps during the experimental procedure, from sample preparation, handling and storage to RNA extraction and preserving, reverse transcription, specific amplification etc [10-12]. Potential experimental inaccuracies can be corrected by normalization of target gene expression to endogenous control as internal reference [13]. Therefore, the selection of proper endogenous control is the first important step in mRNA quantification.

Housekeeping genes are widely used as endogenous controls since their expression is assumed to be stable. However, some studies imply the opposite. It appears that expression of these so called "stable" genes can vary in response to treatment, pathological or environmental conditions, as well as between sexes, tissue types and developmental stages [14-22]. Solving the problem of selection of endogenous control seems to be crucial for experimental design.

The aim of this study was to examine the relation of war trauma and PTSD with the level of expression of four frequently used endogenous controls in order to identify the most stable reference gene for further investigations. Toward that end the expression of β-actin (BA), glyceraldehyde-3-phosphate dehydrogenase (GAPDH), β_2_-microglobulin (B2M) and RNA polymerase II polypeptide A (PolR2A) in PBMCs from war veterans with current or lifetime PTSD, without PTSD (trauma controls), and from healthy non-traumatized subjects was studied by the TaqMan Real-time PCR method.

## Methods

### Subjects

This study was performed on four groups of subjects: (1) war veterans with current PTSD, (2) veterans with PTSD in remission - lifetime PTSD, (3) individuals that experienced war-related traumatic events but did not develop PTSD - trauma controls, and (4) healthy non-traumatized subjects.

Twenty eight subjects participated in the study (7 per group). All participants were male and the groups were matched by age. The traumatic experiences of the first three groups were related to the recent wars in Balkan region.

The inclusion criteria for the subjects with current or lifetime PTSD included: (a) exposure to traumatic war event(s); (b) current PTSD or lifetime PTSD as defined by DSM-IV criteria and assessed by CAPS-DX and (c) CAPS Criteria A-F satisfied, with the score of B+C+D subtotals above 50.

Inclusion criteria for trauma controls were: (a) exposure to traumatic war event(s), (b) no diagnosis of current or lifetime PTSD as defined by the DSM-IV criteria and assessed by the CAPS-DX and (c) CAPS Criteria A-F not fulfilled, score of B+C+D subtotals below 30.

Healthy subjects have never experienced any severe traumatic event and were free from any Axis I disorder.

Exclusion criteria for all groups were: (a) serious medical illness, (b) current psychotic disorder, as defined by DSM-IV criteria (except major depression), (c) current psychoorganic syndrome, as defined by DSM-IV criteria, (d) alcohol dependence or alcohol abuse within 6 months prior to the entry procedure, (e) substance dependence or substance abuse within 6 months prior the entry procedure, and (f) taking medications (list of excluding medications from the groups of benzodiazepines, antidepressants, neuroleptics, antipsychotics) within at least 2 weeks prior to the entry procedure. Structured Clinical Interview for DSM-IV, SCID-CV, was used as a diagnostic tool for comorbidities.

PTSD patients were recruited from Association of Veterans from Wars after 1990, the Association of Ex-detained Persons, and war victims assessed or treated in the NGO International Aid Network, Belgrade, Serbia. Trauma control subjects were recruited from Association of Veterans and from special army forces via Military Medical Academy, Belgrade, Serbia.

Healthy non-traumatized subjects were recruited through available social networks, National Trade Union, and from general population with the assistance of Strategic Marketing Agency. All subjects gave their written informed consent for participating. All aspects of the study were performed in accordance with the ethical standards laid down in the 1964 Declaration of Helsinki and the protocol was approved by the Ethical Committee of the Clinical Center of Serbia.

### Isolation of peripheral blood mononuclear cells

Peripheral blood was obtained by venipuncture. All samples were taken between 08:30 h and 09:30 h. The blood was diluted with an equal volume of PBS (1.5 mM KH_2_PO_4_, 6.5 mM Na_2_HPO_4_, 2.7 mM KCl, 0.14 M NaCl, pH 7.2). PBMCs were prepared from whole blood by Ficoll-Paque PLUS (GE Healthcare Amersham) density gradient centrifugation. Mononuclear cells, recovered from the plasma/Ficoll interface, were extensively washed with PBS and resuspended in RPMI-1640 (Gibco) medium supplemented with 10% heat inactivated fetal calf serum. After centrifugation cell pellets were frozen in liquid nitrogen. Cell viability was assessed by Trypan Blue exclusion, and always found to be more than 95%.

### RNA isolation and cDNA synthesis

Total mRNAs were isolated from previously frozen PBMCs by RNeasy mini kit (Qiagen) according to the manufacturer's instruction. RNA was dissolved in RNase-DNase free water (Eppendorf), RNase inhibitor (Applied Biosystems) added and samples were frozen at -80°C until use. Concentration and purity of RNA were determined spectrophotometricaly (OD 260/280 > 1.8 was considered satisfactory). Integrity of RNA was analyzed by 1% agarose gel electrophoresis. Total RNA was treated with 10 U of RNase free DNase I (Fermentas) according to the manufacturer's protocol.

2 μg of RNA were converted to cDNA by reverse transcription (RT) reactions in a 100 μl volume with random hexamer primers using High-Capacity cDNA Archive Kit (Applied Biosystems) following the manufacturer's instructions. The reactions were carried out under RNase-free conditions at 25°C for 10 min and 37°C for 2 h. Each RT reaction was accompanied by a no RT control in which the reverse transcriptase was replaced by DEPC water. The cDNA was stored at -80°C until further use.

### Real-time PCR

The expression of four commonly used endogenous controls, BA, B2M, GAPDH and PolR2A, were evaluated by TaqMan Real-time RT-PCR. Primers and probes were obtained from Applied Biosystems Assay-on-Demand Gene Expression Products: BA (Hs99999903_m1), B2M (Hs99999907_m1), GAPDH (Hs99999905_m1) and PolR2A (Hs00172187_m1). In order to avoid non-specific product formation, selected TaqMan MGB probes and primers were chosen as to span across exon/exon boundaries. Amplicon lengths were: 171 for BA, 75 for B2M, 122 for GAPDH and 61 bp for PolR2A.

Real-time PCR was performed using ABI Prism 7000 Sequence Detection System (Applied Biosystems) in a total volume of 25 μl containing 1× TaqMan Universal Master Mix with AmpErase UNG, 1× Assay Mix (Applied Biosystems) and cDNA template (20 ng of RNA converted to cDNA) at cycle conditions: 95°C for 10 min, followed by 40 cycles at 95°C for 15 s and 60°C for 90 s. No template control was used in each run. All reactions were run in triplicates.

PCR efficiencies for each assay were derived from standard curves. Serial five-fold dilutions with at least five measuring points of one randomly chosen cDNA were made and amplified, and standard curves constructed. Amplification efficiencies (E%) were calculated, using formula: E = (10^-1/slope ^- 1) × 100.

### Data analysis

Data analysis was performed by direct comparison of Ct values, followed by computer-assisted analysis using software packages: GeNorm 3.5 http://medgen.ugent.be/~jvdesomp/genorm[18] and Normfinder http://www.mdl.dk/publicationsnormfinder.htm[23]. GeNorm makes pairwise comparison between one endogenous control and all other reference genes, in all samples. The software ranks endogenous controls gene stability by average expression stability value (M). NormFinder is an application for Microsoft Excel, which provides information on intra- and inter-group variability, the information regarding best endogenous control as well as best combination of endogenous controls. More stable gene expression is indicated by lower average expression stability values.

## Results

The aim of this study was to identify the most stabile reference gene(s) that could be used for the normalization of quantification of mRNA expression in PBMCs from war veterans with and without PTSD. Using Real-time RT-PCR we evaluated the variability in expression of four frequently used endogenous controls (BA, B2M, GAPDH, PolR2A) in PBMCs from: current PTSD patients, lifetime PTSD patients, trauma controls and healthy subjects. To compare RNA transcription levels of these genes between the four groups, we compared Ct values directly, and also analyzed them using GeNorm and NormFinder.

The first step in our analysis was to test PCR efficiency of four potential endogenous controls by amplifying serial dilutions of one randomly chosen cDNA sample. Amplification efficiencies of candidate endogenous controls were calculated and presented in Table 1. All r^2 ^values of the standard curves exceeded 0.998.

**Table 1:** Gene symbol, molecular function and amplification efficiency of four candidate endogenous controls.

Gene Symbol	Gene Name	Function	Amplification efficiency E (%)
BA	β-Actin	Cytoskeletal structural protein	96
B2M	β_2_-Microglobulin	β-Chain of major histocompatibility complex class I molecule	91
GAPDH	Glyceraldehyde-3-phosphate dehydrogenase	Glycolytic enzyme	103
PolR2A	RNA polymerase II polypeptide A	DNA directed RNA polymerase activity	98

**Figure 1:**
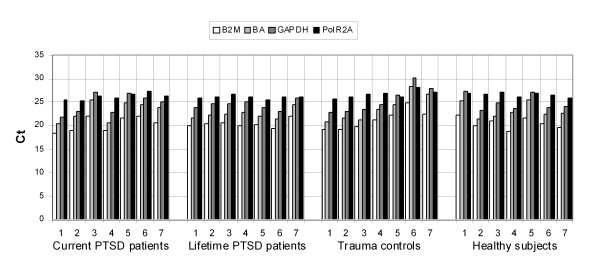
**Expression profiles of four tested endogenous controls**. Ct values of each gene and each individual subject are presented.

Next step was to check the effect of war trauma and/or PTSD on expression stability of four possible endogenous controls. First, to explore the effect of the disease and/or trauma on mRNA level of four examined endogenous controls the individual Ct values in all subjects were compared. Ct value is inversely correlated with the amount of cDNA present in the reaction. Expression profiles of B2M, BA, GAPDH and PolR2A are presented in Figure 1. Abundance of the examined endogenous controls was B2M>BA>GAPDH>PolR2A in almost all subjects. Analysis of Ct values for each gene showed inter-individual variations. Specifically, the range of Ct values was 2.88 for PolR2A, 6.5 for B2M and 8 for GAPDH and BA. Therefore PolR2A showed the lowest variation in Ct values, but BA, GAPDH and B2M showed the same pattern of expression throughout all individuals and conditions. Intra-assay variation was <0.5% and inter-assay variation <1.5% for all assays.

**Figure 2:**
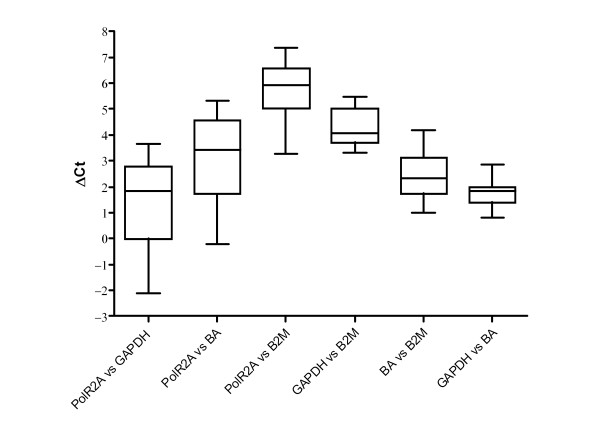
**Comparison of ΔCt values of pairs of potential endogenous controls**. ΔCt values were calculated for each of 28 subjects. Medians are shown as lines, percentiles as boxes and ranges as whiskers.

As the next step, the differences between all possible pairs of Ct values of potential endogenous controls (ΔCt values) were compared between all subjects (Figure 2). The rationale was that if ΔCt values of one pair of endogenous controls remain constant in all 28 subjects, it implies either stable expression or co-regulation. Our results showed that PolR2A-containing pairs had high variability, and assuming no co-regulation, PolR2A should be considered unsuitable endogenous control for trauma-exposed and PTSD subjects. On the other hand, pairs GAPDH *vs *B2M, BA *vs *B2M and GAPDH *vs *BA showed less inter-individual variability, implying more stable expression or possible co-regulation.

**Figure 3:**
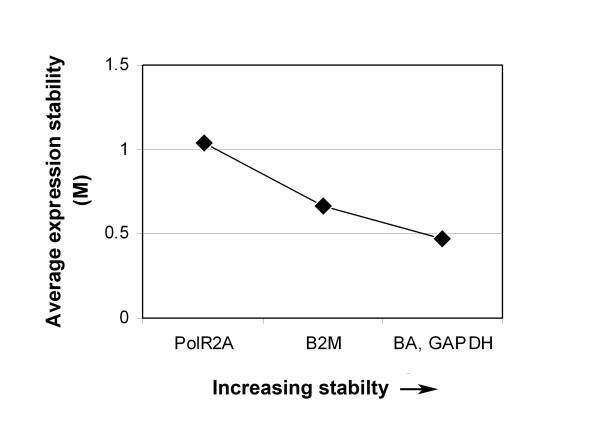
**GeNorm analysis of BA, GAPDH, B2M and PolR2A**. The analysis includes a stepwise exclusion of the least stable endogenous control. Endogenous controls are ranked in order of their expression stability and presented at x-axis. Stability values (M) are presented at y-axis. Low stability value (M) reflects greater stability.

To verify these assumptions, gene expression stability value (M) for B2M, BA, GAPDH and PolR2A in PBMCs of the four groups of subjects was calculated by GeNorm and NormFinder softwares (Table 2). The basic assumption of GeNorm method is that the ratio of two perfect reference genes should be constant under different experimental conditions. Stability value M is calculated for each endogenous control, reflecting the pair-wise variability between one and all the others. The least stable candidate is excluded, and the M value recalculated. GeNorm identified BA and GAPDH as a pair of genes with the lowest stability value, indicating this combination of genes as the most stable one (Figure 3). The individual M values are shown in Table 2. The analysis revealed B2M as the most stable gene, followed by GAPDH and BA, while PolR2A was the least stable one. The GeNorm software also analyzed pair-wise variation values between two sequential normalization factors (geometric means of best reference genes). Normalization factors were calculated by stepwise inclusion of extra, less stable reference gene to determine how many reference genes should be used. A large variation means that the added gene has a significant effect on normalization factor and should be included for calculation. Vandesompele et al [18] propose pair-wise variation of 0.15 as a cut-off under which the inclusion of additional reference gene is not required. The pair-wise variation upon normalization with the two most stable genes in our study and introduction of the third one was 0.246. This value increased to 0.342 after addition of the fourth gene. Since in this study only four reference genes were analyzed, the cut-off of 0.15 was not considered in a strict sense but rather as a guide for selection of reliable set of genes for normalization. The four potential reference genes examined in our study belong to different functional classes, which reduces the chance of co-regulation. Nevertheless, we additionally analyzed exponentially transformed data (2^-ΔCt^) by the NormFinder software. NormFinder estimates both inter- and intra-group variations, and combines both of these in stability value. Similarly to GeNorm, NormFinder pointed to BA and GAPDH combination as the most stable one, with stability value of 0.006. As for the individual genes (Table 2), GAPDH was identified as the most stable gene (stability value 0.008), while PolR2A as the least stable one (stability value 0.039).

**Table 2:** Expression stability values of GAPDH, BA, B2M and PolR2A calculated by GeNorm and NormFinder softwares.

Gene	GeNorm Stability value (M)	NormFinder Stability value (M)
GAPDH	0.898	0.008
BA	0.972	0.010
B2M	0.869	0.009
PolR2A	1.404	0.039

High expression stability corresponds to low stability value (M).

Since the NormFinder provides information on intra- and inter-group variations, we have re-analyzed the results across several subject group combinations. NormFinder software was used to calculate stability values, specify the best reference gene and the best combination of two genes that can be used for future comparisons between various combinations of subject groups (Table 3). B2M was identified as a suitable single endogenous control for comparing changes in gene expression between current PTSD patients and healthy non-traumatized subjects; between current PTSD patients and trauma controls, and between current PTSD patients, trauma controls and healthy non-traumatized subjects (Table 3). GAPDH can be used as a reference gene for gene expression studies designed to compare gene expression in PBMCs from current and lifetime PTSD patients with trauma controls, while to compare changes in gene expression between current PTSD patients, lifetime PTSD patients and healthy subjects, BA can be used as a single endogenous control (Table 3). As the best combinations of two reference genes that can be used for comparisons mentioned above, the software pointed to B2M and GAPDH, and to B2M and BA (Table 3).

**Table 3:** Expression stability values of the best reference gene and the best combination of two genes for between-group comparisons.

	Current PTSD patients *vs*. trauma controls	Current PTSD *vs*. healthy subjects	Current PTSD, trauma controls *vs*. healthy subjects	Current PTSD, lifetime PTSD *vs*. trauma controls	Current PTSD, lifetime PTSD *vs*. healthy subjects
**Best gene**	B2M	B2M	B2M	BA	GAPDH
**Stability value (M)**	0.011	0.037	0.008	0.007	0.029

**Best combination of two genes**	B2M and BA	B2M and GAPDH	B2M and GAPDH	B2M and BA	B2M and GAPDH
**Stability value for best combination of two genes (M)**	0.009	0.041	0.007	0.006	0.029

Stability values were calculated by NormFinder software. High expression stability corresponds to low stability value (M).

## Discussion

This study was designed as to identify suitable reference gene(s) for normalization of gene expression in PBMCs in response to war trauma and PTSD. The expression stability of four possible endogenous controls was investigated in PBMCs from war veterans with current PTSD, those with lifetime PTSD, trauma controls and healthy non-traumatized subjects, in order to find the most suitable reference gene(s) that can be used in the future studies for comparing transcriptional changes between these groups. Moreover, carefully defined criteria for classification of participants into these four groups enabled the selection of endogenous controls that can also be used for comparisons between the various combinations of these groups, and consequently allow more refined comparisons of gene expression that might be used for future studying of vulnerability and resilience to PTSD, as well as studying developing and recovery from PTSD.

Selection of appropriate endogenous control is a critical step in gene expression analysis, especially in situations where differences in mRNA levels are small. In the present study we analyzed the expression stability of four commonly used endogenous controls (BA, B2M, GAPDH and PolR2A) and showed that combination of BA and GAPDH can serve as a suitable normalizing factor for purposes of studying changes in gene expression in PBMCs related to war trauma and current or lifetime PTSD.

BA, GAPDH and B2M are frequently used endogenous controls, although the issue of using these genes for normalization is a constant matter of debate. BA is ubiquitous cytoskeleton protein, GAPDH is important glycolitic enzyme, and B2M is β-chain of major histocompatibility complex class I molecule. However, apart from their basic cellular roles, these proteins also participate in other cellular functions [24-26]. As a result, previous studies pointed to the variability in expression of these housekeeping genes between sexes, tissue types, as well as in response to treatment and pathological or environmental conditions [9,10,15-22]. On the other hand, there is evidence favoring their use as appropriate internal standards, either separately or in various combinations, in a number of carefully defined conditions.

For example, GAPDH showed stable expression and could be used as a reference gene for gene expression studies in human leukocytes [27], reticulocytes [28], hepatocellular carcinoma [29], prostate cancer [30] and heart tissue [31]. Also, GAPDH was one of the most stable reference genes recommended as endogenous control for investigation of chondroprotective agents in human chondrocytes [32], as well as for comparing age-related changes in gene expression in human skeletal muscle [33].

BA was shown to be a suitable endogenous control for gene expression studies in human neutrophils [34], leukocytes [27] and prostate cancer [30], as well as for comparing gene expression in prefrontal cortex of chronic alcoholics and control subjects [35].

B2M, together with 18S rRNA, was validated as a reference gene for investigations in serum stimulated fibroblasts [20], and for examination of the effects of ionizing radiation and chemical exposure on gene expression in human lymphoblastoid cells [36]. It could also be used as an endogenous control in studies of gene expression in human neutrophils [34], skeletal muscle [33] and osteoarthritic articular cartilage [37], as well as in studies of chondroprotective action of curcumin [32].

PolR2A is the largest subunit of RNA polymerase II complex and the main enzyme in mRNA transcription. The use of PolR2A as endogenous control in human tissues and cell lines has been supported by several authors [22,38]. Radonic and coworkers [22] found that PolR2A was a suitable reference gene for a broad range of human tissues. It was also shown to be one of the most stable genes for normalization of gene expression in non-small cell lung cancer [39], in motor cortex of alcoholics and control subjects [35], and in human skeletal muscle [33]. However, PolR2A did not perform well in our study and both NormFinder and GeNorm identified it as the most variable among the examined genes.

In the present study, direct comparison of Ct values showed marked inter-individual variations for all four validated genes. BA, GAPDH and B2M showed similar pattern of expression throughout all individuals and conditions, whereas the lowest range of Ct values for PolR2A was observed (~3 Ct). However, when the expression stability of BA, B2M, GAPDH and PolR2A in PBMCs of PTSD patients and control subjects was evaluated by GeNorm and NormFinder software packages, PolR2A was identified as the most unstable gene. BA, GAPDH and B2M performed well, although the ranking order differed between these two softwares. NormFinder identified GAPDH, while GeNorm identified B2M as a suitable single endogenous control with the most stable expression. These discrepancies are caused by the differences between the approaches. Simple comparison of Ct values reveals "overall expression variation" without taking into the consideration systematic inter-group variation which is critical and could lead to an incorrect interpretation of the results. The NormFinder top ranks the candidates with minimal estimated intra- and inter-group variation, in contrast to the pairwise comparison approach (GeNorm), which tends to select those genes with the highest degree of similarity of the expression profile across the sample set. The latter approach implies that the candidates with minimal expression variation do not necessarily become top ranked. Therefore, a situation in which the sample set consists of four sample subgroups, and all of the candidates but one (PolR2A) show some variation in and between the groups, like in this case, the candidate with the lowest overall variation would be excluded early on in the pairwise comparison approach. Contrary to this, NormFinder would present such a gene with the smallest stability value of all candidates if it had shown no or small inter-group (particularly) and intra-group variation. However, "overall expression variation" of PolR2A is mostly due to inter-group variation and NormFinder identified PolR2A as the most unstable gene, as well.

Taken together, comparison of Ct values only is not enough because this approach does not take into consideration intra- and inter-group variation and when introducing a larger set of samples, the range of Ct variation could dramatically change. Pair-wise comparison approach (GeNorm) is regarded as the authoritative method for the analysis of potential endogenous controls but it ranks genes according to the similarity of their expression profiles, rather than minimal variation. NormFinder, with its account of sample groups and its direct estimation of expression variation, provides even more precise and robust measure of gene expression stability and most importantly candidate co-regulation does not significantly affect the approach.

Therefore, it is strongly urged to introduce more approaches in validation of endogenous controls and the best situation would be when two of them reveal the same results. Both software packages pointed to BA and GAPDH as the best combination of endogenous controls that can be used for normalization of gene expression in PBMCs from patients with current or lifetime PTSD, trauma controls and healthy subjects. In addition, we have re-analyzed results using NormFinder software in order to provide endogenous controls that can be used for particular comparisons between some of the examined groups of subjects. It appeared that B2M, BA and GAPDH, individually or in combinations (specified in Table 3) can also be used in future studies on changes in gene expression related to vulnerability, recovery and resilience to PTSD.

The advantage of using multiple reference genes combination for normalization instead of GAPDH, B2M or BA alone is not clearly evident in this study since the comparison of the stability values of individual endogenous controls and stability values for best combination of two genes have not revealed significant differences. However, introducing larger set of samples might make a difference and so it is strongly recommended to use more than one reference gene for normalization [18] as a way to increase the accuracy of the results and to reach the sensitivity needed for detection of subtle changes in a target gene expression.

To date only two studies used Real-time RT-PCR to examine gene expression in PBMCs from PTSD patients and healthy subjects [3,4]. Namely, to normalize p11 and GR expression in PBMCs from healthy non-traumatized subjects and patients with PTSD, major depressive disorder, bipolar disorder or schizophrenia, Su and coworkers [4] used BA as a reference gene. Yehuda and coworkers [3] chose reference gene from BA, GAPDH, B2M and ribosomal protein large P0, to normalize the amount of FKBP5 mRNA in PBMCs from current PTSD and non PTSD subjects exposed to 9/11 terrorist attack. In concert with the published data, the results presented herein show that BA, GAPDH and B2M could be used either alone or in a combination, for normalization of gene expression in PBMCs from current and lifetime PTSD patients, trauma controls and healthy subjects. The assumption that exposure to war trauma, current PTSD and lifetime PTSD may be related to different gene expression patterns in PBMCs, determined the way of classification of the subjects participating in this study. The applied classification into the four groups enabled us to select internal reference genes for future studies on gene expression in PBMCs from individuals exposed to war trauma who were susceptible or resilient to PTSD, as well as from those with the current disorder and those who recovered.

As a limitation of our study, we should mention that only four commonly used endogenous controls were examined. We selected genes which belong to distinct biological pathways with the intention to avoid possible co-regulation. In addition, only male subjects participated in our study in order to avoid possible differences between sexes.

## Conclusion

In conclusion, the most reliable and precise quantification of gene expression in PBMCs from war veterans with and without PTSD could be achieved by normalization to a combination of two reference genes: GAPDH and BA. Using GAPDH, BA and B2M as individual endogenous controls would provide satisfactory data, while PolR2A could not be recommended.

## Abbreviations

B2M: β_2_-microglobulin; BA: β-actin; CAPS: Clinician Administrated PTSD Scale; GAPDH: Glyceraldehydes-3-phosphate dehydrogenase; PBMC: Peripheral blood mononuclear cells; PolR2A: RNA polymerase II polypeptide A; PTSD: Posttraumatic stress disorder; SCID-I: Structured Clinical Interview for DSM-IV axis I disorders.

## Authors' contributions

JB and NT designed the experiments and wrote the manuscript. JB and DVM performed the experiments and statistical analysis of the results. GM designed the research project, supervized the study and edited the manuscript. JD, IE, SMJ and TP participated in the experimental design, isolated PBMCs and purified RNA, and critically reviewed the manuscript.

All authors contributed to and have approved the final manuscript.

## References

[B1] Segman RH Shefi N Goltser-Dubner T Friedman N Kaminski N Shalev AY Peripheral blood mononuclear cell gene expression profiles identify emergent post-traumatic stress disorder among trauma survivors Mol Psychiatry 2005 10 5 500 513 425 10.1038/sj.mp.4001636 15685253

[B2] Zieker J Zieker D Jatzko A Dietzsch J Nieselt K Schmitt A Bertsch T Fassbender K Spanagel R Northoff H Differential gene expression in peripheral blood of patients suffering from post-traumatic stress disorder Mol Psychiatry 2007 12 2 116 118 10.1038/sj.mp.4001905 17252001

[B3] Yehuda R Cai G Golier JA Sarapas C Galea S Ising M Rein T Schmeidler J Muller-Myhsok B Holsboer F Gene expression patterns associated with posttraumatic stress disorder following exposure to the World Trade Center attacks Biol Psychiatry 2009 66 7 708 711 10.1016/j.biopsych.2009.02.034 19393990

[B4] Su TP Zhang L Chung MY Chen YS Bi YM Chou YH Barker JL Barrett JE Maric D Li XX Levels of the potential biomarker p11 in peripheral blood cells distinguish patients with PTSD from those with other major psychiatric disorders J Psychiatr Res 2009 43 13 1078 1085 10.1016/j.jpsychires.2009.03.010 19380152

[B5] Tang Y Lu A Aronow BJ Sharp FR Blood genomic responses differ after stroke, seizures, hypoglycemia, and hypoxia: blood genomic fingerprints of disease Ann Neurol 2001 50 6 699 707 10.1002/ana.10042 11761467

[B6] Tsuang MT Nossova N Yager T Tsuang MM Guo SC Shyu KG Glatt SJ Liew CC Assessing the validity of blood-based gene expression profiles for the classification of schizophrenia and bipolar disorder: a preliminary report Am J Med Genet B Neuropsychiatr Genet 2005 133B 1 1 5 10.1002/ajmg.b.30161 15645418

[B7] van Heerden JH Conesa A Stein DJ Montaner D Russell V Illing N Parallel changes in gene expression in peripheral blood mononuclear cells and the brain after maternal separation in the mouse BMC Res Notes 2009 2 1 195 10.1186/1756-0500-2-195 19781058 PMC2759952

[B8] Gladkevich A Kauffman HF Korf J Lymphocytes as a neural probe: potential for studying psychiatric disorders Prog Neuropsychopharmacol Biol Psychiatry 2004 28 3 559 576 10.1016/j.pnpbp.2004.01.009 15093964

[B9] Bustin SA Mueller R Real-time reverse transcription PCR (qRT-PCR) and its potential use in clinical diagnosis Clin Sci (Lond) 2005 109 4 365 379 10.1042/CS20050086 16171460

[B10] Bustin SA Quantification of mRNA using real-time reverse transcription PCR (RT-PCR): trends and problems J Mol Endocrinol 2002 29 1 23 39 10.1677/jme.0.0290023 12200227

[B11] Bustin SA Nolan T Pitfalls of quantitative real-time reverse-transcription polymerase chain reaction J Biomol Tech 2004 15 3 155 166 15331581 PMC2291693

[B12] Fleige S Pfaffl MW RNA integrity and the effect on the real-time qRT-PCR performance Mol Aspects Med 2006 27 2-3 126 139 10.1016/j.mam.2005.12.003 16469371

[B13] Huggett J Dheda K Bustin S Zumla A Real-time RT-PCR normalisation; strategies and considerations Genes Immun 2005 6 4 279 284 10.1038/sj.gene.6364190 15815687

[B14] Barber RD Harmer DW Coleman RA Clark BJ GAPDH as a housekeeping gene: analysis of GAPDH mRNA expression in a panel of 72 human tissues Physiol Genomics 2005 21 3 389 395 10.1152/physiolgenomics.00025.2005 15769908

[B15] Derks NM Muller M Gaszner B Tilburg-Ouwens DT Roubos EW Kozicz LT Housekeeping genes revisited: different expressions depending on gender, brain area and stressor Neuroscience 2008 156 2 305 309 10.1016/j.neuroscience.2008.07.047 18722514

[B16] McCurley AT Callard GV Characterization of housekeeping genes in zebrafish: male-female differences and effects of tissue type, developmental stage and chemical treatment BMC Mol Biol 2008 9 102 10.1186/1471-2199-9-102 19014500 PMC2588455

[B17] Thellin O Zorzi W Lakaye B De Borman B Coumans B Hennen G Grisar T Igout A Heinen E Housekeeping genes as internal standards: use and limits J Biotechnol 1999 75 2-3 291 295 10.1016/S0168-1656(99)00163-7 10617337

[B18] Vandesompele J De Preter K Pattyn F Poppe B Van Roy N De Paepe A Speleman F Accurate normalization of real-time quantitative RT-PCR data by geometric averaging of multiple internal control genes Genome Biol 2002 3 7 RESEARCH0034 10.1186/gb-2002-3-7-research0034 12184808 PMC126239

[B19] Verma AS Shapiro BH Sex-dependent expression of seven housekeeping genes in rat liver J Gastroenterol Hepatol 2006 21 6 1004 1008 10.1111/j.1440-1746.2005.03948.x 16724986

[B20] Schmittgen TD Zakrajsek BA Effect of experimental treatment on housekeeping gene expression: validation by real-time, quantitative RT-PCR J Biochem Biophys Methods 2000 46 1-2 69 81 10.1016/S0165-022X(00)00129-9 11086195

[B21] Dheda K Huggett JF Chang JS Kim LU Bustin SA Johnson MA Rook GA Zumla A The implications of using an inappropriate reference gene for real-time reverse transcription PCR data normalization Anal Biochem 2005 344 1 141 143 10.1016/j.ab.2005.05.022 16054107

[B22] Radonic A Thulke S Mackay IM Landt O Siegert W Nitsche A Guideline to reference gene selection for quantitative real-time PCR Biochem Biophys Res Commun 2004 313 4 856 862 10.1016/j.bbrc.2003.11.177 14706621

[B23] Andersen CL Jensen JL Orntoft TF Normalization of real-time quantitative reverse transcription-PCR data: a model-based variance estimation approach to identify genes suited for normalization, applied to bladder and colon cancer data sets Cancer Res 2004 64 15 5245 5250 10.1158/0008-5472.CAN-04-0496 15289330

[B24] Nomura T Huang WC Zhau HE Wu D Xie Z Mimata H Zayzafoon M Young AN Marshall FF Weitzmann MN Beta2-microglobulin promotes the growth of human renal cell carcinoma through the activation of the protein kinase A, cyclic AMP-responsive element-binding protein, and vascular endothelial growth factor axis Clin Cancer Res 2006 12 24 7294 7305 10.1158/1078-0432.CCR-06-2060 17189401

[B25] Percipalle P Visa N Molecular functions of nuclear actin in transcription J Cell Biol 2006 172 7 967 971 10.1083/jcb.200512083 16549500 PMC2063754

[B26] Sirover MA New insights into an old protein: the functional diversity of mammalian glyceraldehyde-3-phosphate dehydrogenase Biochim Biophys Acta 1999 1432 2 159 184 10407139 10.1016/s0167-4838(99)00119-3

[B27] Spinsanti G Zannolli R Panti C Ceccarelli I Marsili L Bachiocco V Frati F Aloisi AM Quantitative Real-Time PCR detection of TRPV1-4 gene expression in human leukocytes from healthy and hyposensitive subjects Mol Pain 2008 4 51 10.1186/1744-8069-4-51 18983665 PMC2588574

[B28] Silver N Best S Jiang J Thein SL Selection of housekeeping genes for gene expression studies in human reticulocytes using real-time PCR BMC Mol Biol 2006 7 33 10.1186/1471-2199-7-33 17026756 PMC1609175

[B29] Cicinnati VR Shen Q Sotiropoulos GC Radtke A Gerken G Beckebaum S Validation of putative reference genes for gene expression studies in human hepatocellular carcinoma using real-time quantitative RT-PCR BMC Cancer 2008 8 350 10.1186/1471-2407-8-350 19036168 PMC2607287

[B30] Mori R Wang Q Danenberg KD Pinski JK Danenberg PV Both beta-actin and GAPDH are useful reference genes for normalization of quantitative RT-PCR in human FFPE tissue samples of prostate cancer Prostate 2008 68 14 1555 1560 10.1002/pros.20815 18651557

[B31] Perez S Royo LJ Astudillo A Escudero D Alvarez F Rodriguez A Gomez E Otero J Identifying the most suitable endogenous control for determining gene expression in hearts from organ donors BMC Mol Biol 2007 8 114 10.1186/1471-2199-8-114 18096027 PMC2234425

[B32] Toegel S Huang W Piana C Unger FM Wirth M Goldring MB Gabor F Viernstein H Selection of reliable reference genes for qPCR studies on chondroprotective action BMC Mol Biol 2007 8 13 10.1186/1471-2199-8-13 17324259 PMC1820791

[B33] Touchberry CD Wacker MJ Richmond SR Whitman SA Godard MP Age-related changes in relative expression of real-time PCR housekeeping genes in human skeletal muscle J Biomol Tech 2006 17 2 157 162 16741243 PMC2291775

[B34] Zhang X Ding L Sandford AJ Selection of reference genes for gene expression studies in human neutrophils by real-time PCR BMC Mol Biol 2005 6 1 4 10.1186/1471-2199-6-4 15720708 PMC551605

[B35] Johansson S Fuchs A Okvist A Karimi M Harper C Garrick T Sheedy D Hurd Y Bakalkin G Ekstrom TJ Validation of endogenous controls for quantitative gene expression analysis: application on brain cortices of human chronic alcoholics Brain Res 2007 1132 1 20 28 10.1016/j.brainres.2006.11.026 17188656

[B36] Banda M Bommineni A Thomas RA Luckinbill LS Tucker JD Evaluation and validation of housekeeping genes in response to ionizing radiation and chemical exposure for normalizing RNA expression in real-time PCR Mutat Res 2008 649 1-2 126 134 17904413 10.1016/j.mrgentox.2007.08.005

[B37] Pombo-Suarez M Calaza M Gomez-Reino JJ Gonzalez A Reference genes for normalization of gene expression studies in human osteoarthritic articular cartilage BMC Mol Biol 2008 9 17 10.1186/1471-2199-9-17 18226276 PMC2248200

[B38] Macpherson JS Jodrell DI Guichard SM Validation of real-time reverse-transcription-polymerase chain reaction for quantification of capecitabine-metabolizing enzymes Anal Biochem 2006 350 1 71 80 10.1016/j.ab.2005.11.040 16438929

[B39] Saviozzi S Cordero F Lo Iacono M Novello S Scagliotti GV Calogero RA Selection of suitable reference genes for accurate normalization of gene expression profile studies in non-small cell lung cancer BMC Cancer 2006 6 200 10.1186/1471-2407-6-200 16872493 PMC1557528

